# Characterization and phylogenetic analysis of the complete chloroplast genome of *Curcuma longa* (Zingiberaceae)

**DOI:** 10.1080/23802359.2019.1664343

**Published:** 2019-09-12

**Authors:** Dong-Mei Li, Chao-Yi Zhao, Ye-Chun Xu

**Affiliations:** Guangdong Key Lab of Ornamental Plant Germplasm Innovation and Utilization, Environmental Horticulture Research Institute, Guangdong Academy of Agricultural Sciences, Guangzhou, China

**Keywords:** Curcuma longa, Zingiberaceae, chloroplast genome, phylogenetic analysis, single-nucleotide polymorphism

## Abstract

*Curcuma longa*, a well-known traditional medicinal plant in China, belongs to the genus *Curcuma* family Zingiberaceae. In this study, we firstly assembled the complete chloroplast genome of *C. longa* based on sequences from Illumina and PacBio sequencing platforms. We obtained the complete chloroplast genome with the total length of 162,176 bp. It consisted of a large single-copy region (LSC, 86,984 bp), a small single-copy region (SSC, 15,694 bp), and a pair of inverted repeats (IRs, 29,749 bp each). Sequence analyses indicated that the chloroplast genome contained 111 distinct genes including 79 protein-coding genes, 28 tRNA genes, and four rRNA genes. The nucleotide composition was asymmetric (31.62% A, 18.42% C, 17.79% G, 32.18% T) with an overall AT content of 63.80%. The AT contents of the LSC, SSC and IR regions were 66.00%, 70.35% and 58.85%, respectively. Sixteen genes owned a single intron, while another two genes had two introns. The phylogenetic analysis indicated that *C. longa* was closely related to species *Curcuma roscoeana* within the genus *Curcuma* in family Zingiberaceae.

*Curcuma longa* Linnaeus is a species of perennial herb within the genus *Curcuma* in family Zingiberaceae, which is widely cultivated as medicinal and spice plant with great economic value in southern to southwestern China and tropical Asia (Wu and Larsen 2000; Wu et al. 2016). The dried rhizomes of *C. longa* are usually used as Chinese medicines in the treatment of various diseases including hypnotic, anti-inflammatory, analgesic, anti-tumor, antibacterial, anti-virus, anti-oxidation, anti-fatigue and so on (Wu et al. 2016). Furthermore, *C. longa* possesses beautiful flowers, and can also be suitable for ornamental plants in courtyard and park (Wu et al. 2016). Previous researches on *C. longa* were mainly focused on its plant characteristics, in vitro micro propagation, natural products, chemistry and biological activities (Wu et al. 2016; Ma and Gang 2006; Park and Kim 2002; Xu et al. 2015), and few studies were conducted on the chloroplast genome of *C. longa*. In this study, we obtained the complete chloroplast genome sequence of *C. longa* by Illumina and PacBio sequencing technologies. The complete chloroplast reported here would be useful for the research on the phylogenetic relationships and conservation of *C. longa*, and species identification within Zingiberaceae.

*C. longa* was collected from Banna, Yunnan province, and stored at the resource garden of environmental horticulture research institute (specimen accession number Cl2015), Guangdong academy of agricultural sciences, Guangzhou, China. Total chloroplast DNA was extracted from about 100 g of fresh leaves of *C. longa* using the sucrose gradient centrifugation method (Li et al. 2012). Chloroplast DNA (accession number ClDNA2017) was stored at −80 °C in Guangdong key lab of ornamental plant germplasm innovation and utilization, environmental horticulture research institute, Guangdong academy of agricultural sciences, Guangzhou, China. Library construction were using Illumina (Illumina, CA, USA) and PacBio (Novogene, Beijing, China) sequencing, respectively.The Illumina and PacBio sequencing data were deposited in the NCBI sequence read archive under accession numbers SRR8189700 and SRR8184506, respectively. After trimming, 72.3 M clean data of 150 bp paired-end reads and 0.95 M clean data of 8–10 kb subreads were generated. The chloroplast genome of *C. longa* was assembled and annotated by using the reported methods (Li, Wu, et al. 2019). The annotated complete chloroplast genome sequence was submitted to the GenBank (accession no. MK262732).

The complete chloroplast genome of *C. longa* was 162,176 bp in length, and comprised a pair of inverted repeat (IR) regions of 29,749 bp each, a large single-copy (LSC) region of 86,984 bp and a small single-copy (SSC) region of 15,694 bp. It was predicted to contain 133 genes in all. The 133 genes included 111 distinct genes consisting of 79 protein-coding genes, 28 tRNA genes, and four rRNA genes; among them, 8 protein-coding genes (*ndhB*, *rpl2*, *rpl23*, *rps7*, *rps12*, *rps19*, *ycf1* and *ycf2*), 8 tRNAs (*trnA-UGC*, *trnH-GUG*, *trnI-CAU*, *trnI-GAU*, *trnL-CAA*, *trnV-GAC*, *trnR-ACG* and *trnN-GUU*), and all 4 rRNAs (*rrn4.5*, *rrn5*, *rrn16* and *rrn23*) repeated in the IR regions. The *rps12* gene was located its first exon in the LSC region and other two exons in the IR regions. In addition, 10 protein-coding genes (*atpF*, *ndhA*, *ndhB*, *rpoC1*, *petB*, *petD*, *rpl2*, *rpl16*, *rps12* and *rps16*) and 6 tRNA genes (*trnK-UUU*, *trnL-UAA*, *trnV-UAC*, *trnI-GAU*, *trnA-UAC* and *trnG-GCC*) had a single intron, while two other genes (*ycf3* and *clpP*) possessed two introns. The nucleotide composition was asymmetric (31.62% A, 18.42% C, 17.79% G, 32.18% T) with an overall AT content of 63.80%. The AT contents of the LSC, SSC and IR regions were 66.00%, 70.35% and 58.85%, respectively.

To obtain its phylogenetic position within the family Zingiberaceae, a molecular phylogenetic tree was constructed using single-nucleotide polymorphism (SNP) arrays from 13 species including *C. longa.* The SNP arrays were obtained as previously described method (Li, Zhao, et al. 2019). For each chloroplast genome, all SNPs were connected in the same order to obtain a sequence in FASTA format. Multiple FASTA format sequences alignments were carried out using ClustalX version 1.81 (Thompson et al. 1997). A maximum likelihood phylogenetic tree (Figure 1) was constructed using the SNPs from 13 chloroplast genomes alignment result with MEGA7 (Kumar et al. 2016). Bootstrap values were estimated based on 1,000 replicates. As shown in the phylogenetic tree (Figure 1), *C. longa* is closely related to species *Curcuma roscoeana* within the genus *Curcuma* in the family Zingiberaceae with available SNPs.

**Figure 1.**
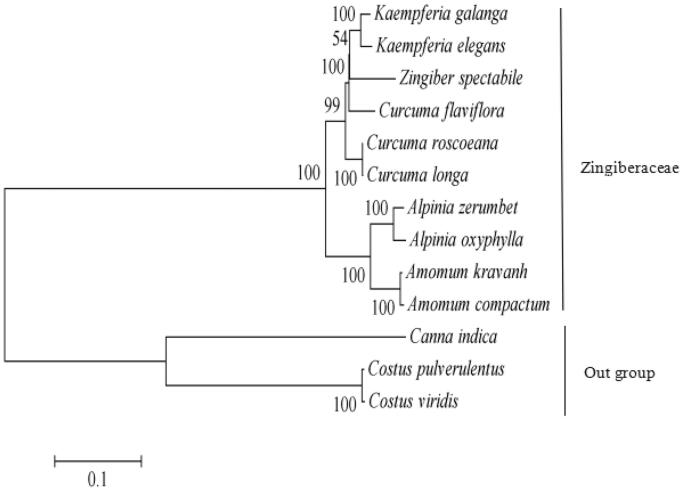
Maximum likelihood tree based on the single-nucleotide polymorphisms (SNPs) among 13 chloroplast genomes. The bootstrap values were based on 1000 replicates and are indicated next to the branches. Accession numbers: *Kaempferia galanga* MK209001, *Kaempferia elegans* MK209002, *Curcuma roscoeana* KF601574, *Curcuma flaviflora* KR967361, *Curcuma longa* MK262732, *Zingiber spectabile* JX088661, *Alpinia zerumbet* JX088668, *Alpinia oxyphylla* KY985237, *Amomum kravanh* MF991963, *Amomum compactum* MG000589, *Canna indica* KF601570, *Costus pulverulentus* KF601573 and *Costus viridis* MK262733.
